# Malignant tumors affecting the head and neck region in ancient times: Comprehensive study of the CRAB Database

**DOI:** 10.1590/1807-3107bor-2024.vol38.0014

**Published:** 2024-01-05

**Authors:** Patricia Verónica AULESTIA-VIERA, Carla Isabelly RODRIGUES-FERNANDES, Thaís Bianca BRANDÃO, André Caroli ROCHA, Pablo Agustin VARGAS, Marcio Ajudarte LOPES, Newell Walter JOHNSON, Luiz Paulo KOWALSKI, Ana Carolina Prado RIBEIRO, Alan Roger SANTOS-SILVA

**Affiliations:** (a) Universidade de São Paulo – USP, Medical School, Clínicas Hospital, São Paulo, SP, Brazil.; (b) Universidade Estadual de Campinas – Unicamp, Piracicaba Dental School, Oral Diagnosis Department, Piracicaba, SP, Brazil.; (c) Universidade de São Paulo – USP, School of Medicine, Dental Oncology Service, São Paulo, SP, Brazil.; (d) Griffith University, Queensland and School of Medicine, Queensland, Australia.; (e) Universidade de São Paulo – USP, Medical School, Head and Neck Surgery Department, São Paulo, SP, Brazil.

**Keywords:** Head and Neck Neoplasms, Paleopathology, Forensic Anthropology, Forensic Pathology, Neoplasms

## Abstract

In the modern world, cancer is a growing cause of mortality, but archeological studies have shown that it is not exclusive to modern populations. The aim of this study is to examine the epidemiologic, social, and clinicopathologic features of head and neck cancers in ancient populations. To do this, we extracted all records that described malignant lesions in the head and neck region available in the Cancer Research in Ancient Bodies Database (CRAB). The estimated age, sex, physical condition of the remains (skeletonized, mummified), anatomic location of tumors, geographic location, chronology, tumor type, and methods of tumor diagnosis were collected. One hundred and sixty-seven cases were found, mostly originating from Europe (51.5%). Most records were of adults between 35 and 49 years of age (37.7%). The most involved site was the skullcap (60.4%), and the most common malignancies were metastases to the bone (65.3%) and multiple myeloma (17.4%). No primary soft tissue malignancies were registered. The results of our study indicate that head and neck cancers were present in ancient civilizations, at least since 500,000 BCE. The available data can help to improve the current understanding of the global distribution of head and neck cancer and its multidimensional impacts on populations in the contemporary world.

## Introduction

Annual deaths due to malignant neoplasms worldwide have continuously increased, from an estimated 6.2 million deaths in 2000 to 9.96 million in 2020.^1-5^ This increase reflects both population growth and the increasing lifespan of many populations, as well as changes in the prevalence and distribution of etiological factors, many of which are associated with socioeconomic level.^2^ Among the most relevant factors are outdoor and indoor air and water pollution, sedentarism, diets low in antioxidant vitamins and minerals, tobacco and areca nut use, sunlight exposure, viruses (e.g., human T-cell leukemia virus type 1, “high-risk” types of human papillomaviruses, hepatitis viruses, Epstein–Barr virus, human herpesvirus 8), and shifts in the bacterial and fungal microbiota.^2,6-8^

Today, the term “head and neck cancer” refers to a heterogeneous group of mainly epithelial neoplasms arising in the mucous membranes of the lip, oral cavity, nasopharynx and paranasal sinuses, oropharynx, hypopharynx, larynx, and salivary glands. Taken together, these neoplasms represent a significant cause of morbidity and mortality. According to the World Health Organization, there were an estimated 19.2 million new cancer cases worldwide in 2020, of which 0.93 million (4.8%) were head and neck cancers. More than 467 thousand deaths were caused by head and neck cancers (4.7% of all cancer deaths) in 2020,^4^ compared to 262 thousand deaths recorded in 2002 (3.9% of all cancer deaths).^9^

Understanding the evolution of malignant neoplasms of the head and neck over human history could shed light on the way etiological factors and pathogenic mechanisms have changed. This knowledge could help improve cancer prevention, diagnosis and care. Archeological studies have shown that cancer is not just a disease of modern societies but has been a health hazard to populations since at least the Neolithic period.^10-12^

Primordial humans differed significantly from modern humans in terms of biology, cultural capabilities and social organization. Natural environmental carcinogens, such as sunlight exposure, and indoor pollutants, including smoke from wood fires, must have affected our ancestors. Genetic changes would have also caused biological variations over time, including in respect of cancer susceptibility, and sociocultural changes would have altered the characteristics of human neoplasms.^13,14^

Since the discovery of the “Kanam mandible”, which may be the earliest known example of a malignant neoplasm in a human being dating to approximately 1.5 million years ago in Africa, scientists have focused on understanding the natural history of cancer.^13^ In 2004, Halperin introduced the term paleo-oncology, to refer to a branch of paleopathology (the study of ancient disease) that studies cancer in ancient populations and seeks information about the possible influences of morphologic, functional evolutionary and environmental factors.^15^

Apart from one previous systematic review, bioarcheological and paleopathological sources have mostly reported single cases of cancer, impairing the assemblage and analysis of demographic, spatial, and tumor-related data that could lead us to an improved understanding of the etiology, epidemiology and natural history of this disease.^16^ This study aims to describe the epidemiologic, social, and clinicopathologic features head and neck cancer cases in ancient populations using the records compiled in a paleo-oncological database.

## Methodology

This study was conducted from February to October 2021. All data were retrieved from the Cancer Research in Ancient Bodies Database (CRAB).^17^ This database was developed by the Paleo-oncology Research Organization^18^ and is a compilation of information regarding the published evidence of cancer in the skeletal or soft tissue remains of humans who lived before the 1900s - Common Era (CE) and includes peer-reviewed articles, bioarcheological reports, conference papers, and reference books. The database was accessed in February 2021 (no data update was observed until May 2023). The inclusion criteria were: a) the presence of a malignant lesion as the first diagnostic hypothesis; b) the lesion involved the head and neck region (skull vault, skull base, maxillofacial bones, nasal cavity, paranasal sinuses, nasopharynx, parapharyngeal space, oral cavity, oropharynx, neck and its lymph nodes, salivary glands, and ear; and c) metastatic tumors in the maxillofacial and cranial bones, multiple myeloma (MM), head and neck lesions due to leukemia and malignant meningiomas. The exclusion criteria were: benign tumors, malignant tumors diagnosed outside the head and neck region, and unknown diagnoses.

The estimated age, sex and the physical condition of the remains (skeletonized, mummified), anatomic location of tumors, geographic location, chronology, tumor type, and methods of tumor diagnosis were collected. The original records were consulted if the data were unclear or incomplete in the database. For chronological analyses, the notations Before Common Era (BCE) and Common Era (CE) of the Gregorian calendar were used. The individuals’ ages were categorized according to Buikstra and Ubelaker’s standard age categories, based on mean age-at-death.^19^ When age could not be determined between two adjacent age categories, the inferior range was considered. Data collection and organization were performed using Microsoft Office Excel 2021 software^â^ (Microsoft Corporation, Redmond, USA) and further analyzed by descriptive statistics using absolute numbers and percentages with the same software.

## Results

**Figure 1:**
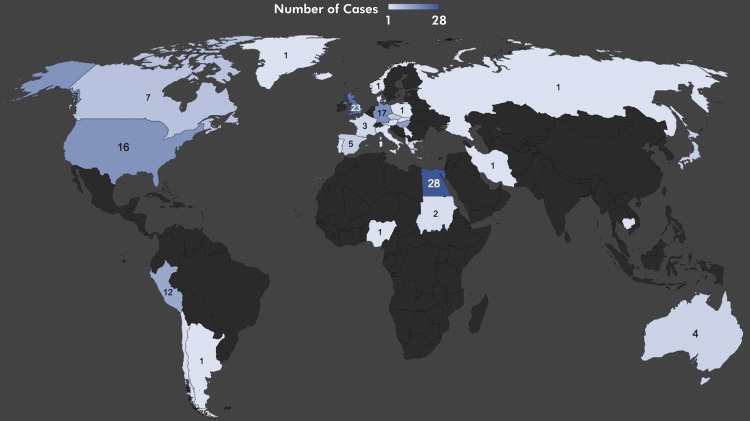
Geographic distribution of head and neck malignant lesion cases by country.

**Figure 2:**
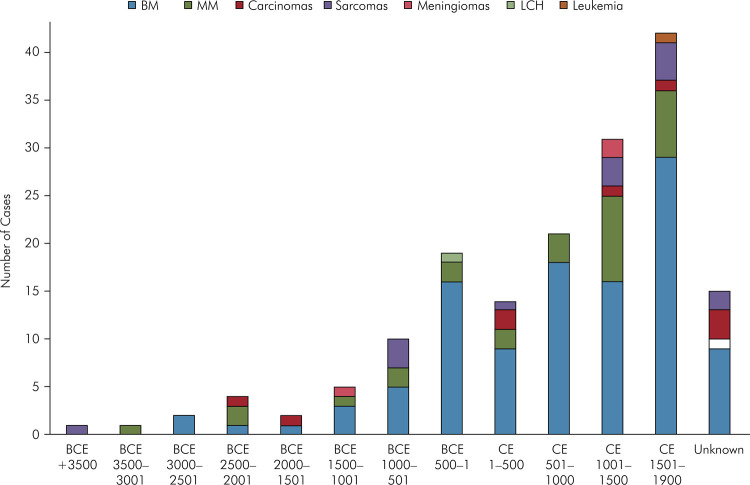
Chronology based on the estimated date the individuals lived (according to the type of cancer). BCE: before common era; CE: common era; LCH: Langerhans cell histiocytosis.

The search resulted in 275 records of malignant tumors published between 1909 and 2017, of which 167 (60.7%) were in the head and neck region. Most human remains were skeletonized (155; 92.8%). Eleven cases (6.6%) of mummies with head and neck injuries were found.^20^ In one case (0.6%) from Egypt, it was unclear whether the body was mummified or skeletonized.^21^ Most of the human remains were found in Europe (86 remains; 51.5%), followed by Africa (31 remains; 18.6%) and North America (23 remains; 13.8%). The countries with the leading number of cases were Egypt (28 cases; 16.8%), the United Kingdom (23 cases; 13.8%), and Germany (17 cases; 10.2%) (Figure 1). The earliest time registered for a case showing a malignant tumor (osteosarcoma) was in skeletal remains estimated to be from more than 500,000 years BCE.^22^ The largest number of human remains found were from 1,501–1,900 CE (42 remains; 25.2%), followed by remains from 1,001–1,500 CE (31; 18.6%). Figure 2 shows the chronology based on the estimated date the individuals lived, presented according to the type of cancer. Seventy-nine of the patients were males (47.3%) and 64 were females (38.3%); in 24 records, sex was not determined (14.4%). Cancers of the head and neck region were most frequently described among adults between 35 and 49 years of age (63 individuals; 37.7%), followed by older adults aged > 50 years (40 individuals; 24%). Moreover, 24 and six individuals were young adults and children, respectively (14.4%; 3.6%). The age was undetermined in 34 cases (20.4%). Table 1 shows the patterns of sex and age distribution according to the type of malignancy.

**Table 1:** Sex and age distribution according to the type of malignancy.

Variables	BM	MM	CA	SAR	Other	Total

	n (%)	n (%)	n (%)	n (%)	n (%)	n (%)
Age* (years)						
Nonadult - 0–19	0 (0.0)	1 (3.4)	0 (0.0)	3 (20.0)	2 (40.0)	6 (3.6)
Young Adult - 20–34 y	15 (13.8)	3 (10.3)	1 (11.1)	5 (33.3)	0 (0.0)	24 (14.4)
Middle Adult - 35–49 Y	41 (36.7)	15 (51.7)	2 (22.2)	4 (26.7)	1 (20.0)	63 (37.7)
Old Adult - ≥ 50	29 (26.6)	7 (25.1)	3 (33.3)	0 (0.0)	1 (20.0)	40 (24.0)
Unknown Age	24 (22.0)	3 (10.3)	3 (33.3)	3 (20.0)	1 (20.0)	34 (20.4)
Sex						
Male	51 (46.8)	15 (51.7)	5 (55.6)	6 (40.0)	2 (40.0)	79 (47.3)
Female	46 (42.2)	12 (41.4)	2 (22.2)	3 (20.0)	1 (20.0)	64 (38.3)
Indeterminate	12(11.0)	2 (6.9)	2 (22.2)	6 (40.0)	2 (40.0)	24 (14.4)

*Age distribution according to Buikstra and Ubelaker’s Standards. BM: bone metastases; MM: multiple myeloma; CA: carcinoma; SAR: sarcoma.

The topographic distribution is summarized in Table 2. The skullcap was predominantly involved (139 cases; 60.4%), followed by the mandible (32 cases; 13.9%). Fifty cases (29.9%) affected more than one site. In total, 230 anatomic subsites were involved in the 167 bodies. In 75 body remains (44.9%), postcranial lesions were described beyond the head and neck tumors, of which 57 cases belonged to the bone metastasis group (BM) [76%], 13 cases belonged to the MM group (17.3%), and three were sarcomas (4%). BM was the most frequent tumor diagnosed (109 cases; 65.3%). Multiple myeloma comprised 29 cases (17.4%), whereas sarcomas and carcinomas affected 15 and 9 individuals, respectively (9%; 5.4%). Less frequent neoplasms were leukemia (1 case; 0.6%) and Langerhans cell histiocytosis (1 case; 0.6%). Table 3 shows the primary tumors related to bone metastases in the head and neck region. The most prevalent primary tumor originated from the breast (12 cases; 11.0%), followed by melanoma (9 cases; 8.3%); however, the primary site for these melanomas was not specified in the database or in the primary article. Table 4 presents the tumor subtypes and the diagnostic methods applied. Macroscopic investigation was the primary diagnostic tool in all cases, and microscopy was used to refine diagnoses in 29 cases (17.4%). Imaging (by X-ray, computed tomography and/or magnetic resonance) was used for 77 cases (46.1%), and biomolecular tests were used (immune assays) in only one case of MM (0.6%).

**Table 2:** Anatomical distribution of head and neck cancers according to the CRAB database.

Anatomic site	BM	MM	CA	SAR	Other	Total

	n (%)	n (%)	n (%)	n (%)	n (%)	n (%)
Maxillofacial bone						
Mandible	17 (53.1)	9 (28.1)	1 (3.1)	3 (9.4)	2 (6.3)	32 (13.9)
Maxilla	2 (13.3)	4 (26.7)	2 (13.3)	5 (33.3)	2 (13.3)	15 (6.5)
Zygomatic region	3 (42.9)	1 (14.3)	1 (14.3)	2 (28.6)	0 (0.0)	7 (3.0)
Skullcap	101 (72.7)	27 (19.4)	1 (0.7)	6 (4.3)	4 (2.9)	139 (60.4)
Temporal region	9 (100)	0 (0.0)	0 (0.0)	0 (0.0)	0 (0.0)	9 (3.9)
Occipital region	2 (66.7)	0 (0.0)	0 (0.0)	1 (33.3)	0 (0.0)	3 (1.3)
Retroorbital region	2 (100)	0 (0.0)	0 (0.0)	0 (0.0)	0 (0.0)	2 (0.9)
Nasal cavity, paranasal sinuses and skull base						
Skull base	5 (38.5)	2 (15.4)	3 (23.1)	2 (15.4)	1 (7.7)	13 (5.7)
Maxillary sinus	1 (100)	0 (0.0)	0 (0.0)	0 (0.0)	0 (0.0)	1 (0.4)
Nasal cavity	2 (28.6)	1 (14.3)	4 (57.1)	0 (0.0)	0 (0.0)	7 (3.0)
Nasopharynx						
Nasopharynx	0 (0.0)	0 (0.0)	1 (100)	0 (0.0)	0 (0.0)	1 (0.4)
Oral cavity and mobile tongue						
Buccal mucosa	0 (0.0)	0 (0.0)	0 (0.0)	1 (100)	0 (0.0)	1 (0.4)

230 anatomic sites were involved in the 167 bodies. BM: bone metastases; MM: multiple myeloma; CA: carcinoma; SAR: sarcoma.

**Table 3:** Primary tumors related to bone metastases in the head and neck region and its diagnostic methods.

Primary tumor (n [%])	Macroscopy	Microscopy	Imaging	Biomolecular

	n (%)	n (%)	n (%)	n (%)
Breast (12 [11·0])	12 (100)	2 (16.7)	4 (33.3)	0 (0.0)
Bronchiogenic (5 [4.6])	5 (100)	2 (40.0)	3 (60.0)	0 (0.0)
Cervical (1 [0.9])	1 (100)	0 (0.0)	1 (100)	0 (0.0)
Hemangioendothelioma (1 [0.9])	1 (100)	0 (0.0)	0 (0.0)	0 (0.0)
Melanoma (9 [8.3])	9 (100)	9 (100)	0 (0.0)	0 (0.0)
Neck (1 [0.9])	1 (100)	0 (0.0)	1 (100)	0 (0.0)
NPC (2 [1.8])	2 (100)	0 (0.0)	1 (50.0)	0 (0.0)
Oral (1 [0.9])	1 (100)	0 (0.0)	0 (0.0)	0 (0.0)
Prostate (8 [7.3])	8 (100)	4 (50.0)	6 (75.0)	0 (0.0)
Unknown (69 [63.3])	69 (100)	7 (10.2)	35 (50.7)	0 (0.0)
Total (109 [100])	109 (100)	24 (22.0)	51 (46.8)	0 (0.0)

BM: bone metastases; NPC: nasopharyngeal carcinoma.

**Table 4:** Tumor type and subtypes and its diagnostic methods.

Tumor type (n [%])	Macroscopy	Microscopy	Imaging	Biomolecular

	n (%)	n (%)	n (%)	n (%)
Bone metastases (109 [100])	109 (100)	24 (22.0)	51 (46.8)	0 (0.0)
Multiple myeloma (29 [100])	29 (100)	2 (6.9)	19 (65.5)	1 (3.5)
Carcinomas (9 [100])	9 (100)	0 (0.0)	1 (11.1)	0 (0.0)
NPC (8 [88.9])	8 (100)	0 (0.0)	1 (12.5)	0 (0.0)
Gingival carcinoma (1 [11·1])	1 (100)	0 (0.0)	0 (0.0)	0 (0.0)
Sarcomas (15 [100])	15 (100)	2 (13.3)	6 (4.0)	0 (0.0)
Osteosarcoma (11 [73.3])	11 (100)	1 (9.1)	5 (45.5)	0 (0.0)
Ewing’s sarcoma (1 [6.7])	1 (100)	0 (0)	1 (100)	0 (0.0)
Rhabdomyosarcoma (1 [6.7])	1 (100)	1 (100)	0 (0)	0 (0.0)
Paget’s sarcoma (1 [6.7])	1 (100)	0 (0)	0 (0)	0 (0.0)
Sarcoma (1 [6.7])	1 (100)	0 (0)	0 (0)	0 (0.0)
Leukemia (1 [100])	1 (100)	0 (0.0)	0 (0.0)	0 (0.0)
Langerhans Cell Histiocytosis (1 [100])	1 (100)	0 (0.0)	0 (0.0)	0 (0.0)
Meningioma – Malignant (3 [100])	3 (100)	1 (33.3)	1 (33.3)	0 (0.0)
Total (167 [100])	167 (100)	29 (17.4)	77 (46.1)	1 (0.6)

## Discussion

Over the last two decades, some scientists have argued that cancer rates in the past were similar to those seen among modern societies; conversely, other researchers have stated that malignant tumors were extremely rare in ancient populations, indicating that cancer is a disease of modernity.^14^ However, we do not know if the prevalence of cancer was really low in antiquity or if many cases were simply not detected. Several factors may explain this lack of evidence of cancer in early populations: a) life expectancy was shorter; b) numerous modern chemical and physical carcinogens did not exist; c) archeological findings mainly consist of skeletal remains; thus, primary soft tissue tumors are almost impossible to identify; d) in the past, many soft tissue malignancies went untreated and may have killed the patient before sufficient time had progressed for the occurrence of bone metastases; e) bone metastases appear first in the marrow cavity, and a superficial inspection of skeletons may fail to reveal inner growths unless radiology is employed; f) the study of ancient tumors depends on the state of preservation of the human remains; and g) malignant tumors may have been overlooked by anthropologists with little medical training.^23,24^

Although establishing the actual prevalence of any neoplastic condition in ancient civilizations is impossible,^14,25^ the evidence considered in the present study does indicate that cancers of the head and neck region have been present in human societies for thousands of years. Among the malignant tumors found in skeletal remains recorded in the CRAB database, head and neck neoplasms account for 60.7% of all the recorded cases; however, this may not represent a true estimate of the occurrence of malignant tumors in the head and neck compared to other cancers, but may simply reflect the fact that a greater number of skulls are found in a good state of preservation in relation to other parts of the body, especially soft tissues. Additionally, the number of malignancies identified is relatively small, making it difficult to make any meaningful comparisons with the modern day.

Currently, the risk factors linked to some cancers of the head and neck region, especially oral cancer, are well established and tobacco consumption in the form of smoked and smokeless tobacco products, areca nut consumption, heavy alcohol use, and infection with certain oncogenic subtypes of the human papillomavirus (HPV – especially in respect of oropharyngeal cancers) and Epstein-Barr virus (EBV – especially in respect of nasopharyngeal cancers).^10,26^ However, are these risk factors exclusively present in modern societies? Evidence shows that the inhabitants of North America were using tobacco approximately 12,300 years ago^27^ and that this practice had significant spiritual meaning many years before Europeans arrived.^28^ In addition to the effects of tobacco and other plant smoke, these ancient populations are likely to have been exposed to smoke inhalation from wood and coal fires and fat-fed lamps. Alcohol consumption has also been observed since ancient times; Ancient Egyptian inscriptions and documents clearly indicate that beer was a basic dietary staple.^29^ A fermented beverage comparable with beer has been found in 13,000 -year-old stone mortars in Israel,^30^ and wine is well known to have been a popular beverage in Greek and Roman civilizations.^31^

Infectious risk factors may also have been prevalent in the past. Although the role of human papillomavirus (HPV) infection in disease was first elucidated in the 19th century, several sources indicate that the role of HPV infections was recognized early on. The oldest discovered HPV infection was seen in an ancient Egyptian mummy dated to 2400 BCE who presented with HPV-related rectal adenocarcinoma. Additionally, during the Greco-Roman historical period, *Condylomata acuminata*, or venereal warts, were identified and recognized as infectious diseases.^32^ The same could be stated for the Epstein-Barr virus, which has been identified in Andean South American mummies.^33^ The presence of all these risk factors in ancient civilizations could indicate that the current rise in tumor frequencies in present populations may simply be related to a longer life expectancy. However, the vast majority of cases identified in the CRAB database were bone metastases (65.3%) or multiple myeloma (17.4%), which are not related to these modern head and cancer risk factors.

Of the adults whose sex could be determined, most were males. This finding may be because males were exposed to more carcinogens, but it may also be the result of greater resistance to decomposition for male bones.^34^ Data concerning age at death are often unavailable or imprecise; however, children were mostly affected by sarcomas, whereas middle-aged and older adults were mostly affected by bone metastases and multiple myeloma, which is consistent with current epidemiological patterns, where these latter malignancies are more frequent in older adults individuals.

Concerning geographical distribution, possible cases of ancient head and neck cancers were identified in six continents (Africa, Asia, Europe, North America, South America, and Australia) and in at least 32 countries. Certain regions (Egypt and the Andean countries) are particularly well represented, which may be the result of differential preservation (mummification being an excellent methodology of organic preservation in archaeology).^14^ Other differences in the number of head and neck cancers found in different parts of the world may be explained by a different incidence rate of a certain cancer in a particular region, similar to what is currently known about certain types of cancer, such as lip and oral cavity cancers, which are highly prevalent in Southern Asia and the Pacific Islands, and nasopharyngeal cancer, which has a higher incidence in North Africa than in other parts of the world;^2,24^however, specific information about ethnicity was not available in the CRAB database. Other differences in the prevalence of head and neck cancers may simply be the result of greater archeological activity in one area than in another.

Our analysis of the CRAB database showed that sarcomas and carcinomas affected 9% and 5.4% of the individuals, respectively. This rate differs from modern incidence rates, where sarcomas are less prevalent than carcinomas.^4^ Eight of the nine cases of carcinomas were nasopharyngeal, most of which were found in Egypt, consistent with the higher prevalence of nasopharyngeal cancer in North African countries.^24^ Bone metastasis (BM) was the most frequent tumor diagnosed (109 cases; 65.3%) in the CRAB database, especially in older individuals. Metastatic bone disease is much more common than primary malignant bone neoplasms, and they consequently will be more frequently encountered in archeological human skeletal remains.^35^ In paleo-oncology, the differentiation of metastatic lesions in skeletal remains is difficult, and sometimes impossible, as they often present very similar skeletal manifestations irrespective of the primary site of the tumor. The CRAB database analysis suggested that the most prevalent primary tumor originated from the breast (12 cases; 11.0%), followed by melanoma (9 cases; 8.3%) and prostate cancer (8 cases; 7.3%). Currently, tumors arising in the breast and prostate gland are known to be particularly likely to disseminate to bone.^12^ Melanomas also often lead to bone metastatic disease; however, 70–86% of these metastases are typically found in the axial skeleton (especially the spine and ribs), whereas the modern metastatic rate in the cranium is only approximately 9%.^36^ In our study, 9 cases of bone metastases in the skull from melanomas were found. These cases were all found in 9 Inca mummies that, according to the authors’ descriptions, presented the majority of osseous lesions on the skull and extremities.^20^ In recent years, the diagnosis of melanoma with bone metastases in these Peruvian mummies has been questioned due to the improbably high prevalence of this tumor in the same region, the infrequency of melanoma in melanodermic ethnic groups who inhabit these regions, the patterns of the bone lesions and the lack of histological confirmation.^36^ Although the CRAB database confirmed that histological examination was used as a diagnostic tool, the authors of the case series stated that in histopathologic sections of the bones, the decalcifying fluid damaged soft tissues, leaving intertrabecular (marrow) spaces empty.^20^

Cases of MM comprised 17.4% of malignant lesions found in the head and neck skeletal remains. Distinguishing between metastatic lesions from carcinomas and melanomas and osseous changes due to hematopoietic neoplasms, such as multiple myeloma, is challenging. These two types of neoplasms cause similar morphological changes in bone, making differential diagnosis impossible in some cases. The absence of associated new bone formation and a tendency toward a more regular size of the osteolytic lesions in multiple myeloma may aid in differentiating these conditions.^37^

Most cases that comprise our sample are represented by skeletal remains with no possibility of soft tissue analysis. In the literature, very little has been documented about paleopathology in soft tissues. A few cases of ancient human neoplasia have been observed in mummified and skeletal remains where the soft tissue tumors underwent calcification. However, the only confirmed case of malignant soft tissue neoplasm in the head and neck region was detected in a male child mummy from northern Chile that presented a lesion in the right cheek. Histological sections showed pleomorphic, disintegrated cells surrounded by fibrous stroma, suggesting rhabdomyosarcoma.^24^ The correct diagnosis of neoplastic lesions often depends on the careful histology of tissue components in addition to the clinical, radiologic, and molecular datasets available in modern oncology. This information will typically not be available to those engaged in the differential diagnosis of neoplasms in archeological human remains,^35^ as we have seen in the CRAB database, and most diagnoses were elucidated only from macroscopic analysis (detection of abnormal bone and its characteristics: osteolytic and /or osteoblastic lesions, size, shape and distribution). However, in older archeological reports there was no standardization of the terminology used among the authors. In some instances, the later re-examination of supposedly neoplastic lesions with the use of modern tools, such as computed tomography, nuclear magnetic resonance, and microscopic and molecular analysis, may allow paleo-oncologists to identify previously undiscovered cases of cancer in the head and neck region or discard cases with misdiagnoses.^14,15^

The findings of this study have to be seen in light of some limitations. First, basing this study on a database means we are relying on the accuracy of others when they entered the data. Second, some publications in the database date back to 1909, when the understanding of neoplasia was very different from today. Therefore, we would currently use different terminology in respect of some of the diagnoses. Finally, although mummies are able to demonstrate a range of head and neck cancer, the majority of cases in the database were based on skeletal remains which only show primary malignancies of bone, local spread from soft tissue malignancy and metastases. This means that some results may be skewed by the type of remains that survive in a region, rather than representing true geographic variation in ancient cancers.

## Conclusion

This article showed that head and neck cancers were present in ancient civilizations, at least since 500,000 BCE. The available data can improve the current understanding of the global distribution of head and neck cancer. However, it is difficult to make direct comparisons with contemporary head and neck malignancies because the small number of ancient human remains may not be representative of the epidemiological reality of those civilizations as no soft tissue primary malignancies were evaluated.
